# High Frequencies of Caspase-3 Expressing *Mycobacterium tuberculosis*-Specific CD4^+^ T Cells Are Associated With Active Tuberculosis

**DOI:** 10.3389/fimmu.2018.01481

**Published:** 2018-06-25

**Authors:** Toidi Adekambi, Chris C. Ibegbu, Stephanie Cagle, Susan M. Ray, Jyothi Rengarajan

**Affiliations:** ^1^Emory Vaccine Center, Emory University School of Medicine, Atlanta, GA, United States; ^2^Department of Microbiology and Immunology, Emory University School of Medicine, Atlanta, GA, United States; ^3^Division of Infectious Disease, Department of Medicine, Emory University School of Medicine, Atlanta, GA, United States

**Keywords:** tuberculosis, caspase-3^+^IFN-γ^+^CD4^+^ T cells, active tuberculosis, latent Mtb infection, anti-tuberculosis treatment, monitoring

## Abstract

Antigen-specific CD4^+^ T cell responses to *Mycobacterium tuberculosis* (Mtb) infection are important for host defense against tuberculosis (TB). However, Mtb-specific IFN-γ-producing T cells do not distinguish active tuberculosis (ATB) patients from individuals with asymptomatic latent Mtb infection (LTBI). We reasoned that the immune phenotype of Mtb-specific IFN-γ^+^CD4^+^ T cells could provide an indirect gauge of Mtb antigen load within individuals. We sought to identify immune markers in Mtb-specific IFN-γ^+^CD4^+^ T cells and hypothesized that expression of caspase-3 Mtb-specific CD4^+^ T cells would be associated with ATB. Using polychromatic flow cytometry, we evaluated the expression of caspase-3 in Mtb-specific CD4^+^ T cells from LTBI and ATB as well as from ATB patients undergoing anti-TB treatment. We found significantly higher frequencies of Mtb-specific caspase-3^+^IFN-γ^+^CD4^+^ T cells in ATB compared to LTBI. Caspase-3^+^IFN-γ^+^CD4^+^ T cells were also more activated compared to their caspase-3-negative counterparts. Furthermore, the frequencies of caspase-3^+^IFN-γ^+^CD4^+^ T cells decreased in response to anti-TB treatment. Our studies suggest that the frequencies of caspase-3-expressing antigen-specific CD4^+^ T cells may reflect mycobacterial burden *in vivo* and may be useful for distinguishing Mtb infection status along with other host biomarkers.

## Introduction

Tuberculosis (TB) is one of the world’s major causes of illness and mortality (1) with about 10 million new cases and 2 million deaths occurring each year. Approximately 10% of individuals infected with *Mycobacterium tuberculosis* (Mtb) develop active TB (ATB), while 90% have no overt signs of clinical disease and are considered to have latent Mtb infection (LTBI) (2) indicating that the host immune response is capable of controlling infection. Several studies have shown that the majority of individuals infected with Mtb mount robust antigen-specific CD4^+^ T cell responses involving T helper 1 (Th1) cytokines, such as IFN-γ and TNF-α, which are critical for activating macrophages and containing bacteria in the lung. However, Th1 cytokines are not sufficient for protection against ATB disease and Mtb-specific IFN-γ-producing CD4^+^ T cells are present in individuals with ATB disease as well as in asymptomatic individuals with LTBI. Moreover, Mtb-specific IFN-γ-producing T cells fail to discriminate between active and LTBI (3, 4) and are not useful for assessing response to active TB treatment, which is typically monitored by sputum culture conversion (5, 6).

In an effort to identify biomarkers in human peripheral blood mononuclear cells (PBMCs) that distinguish active and LTBI states, we previously characterized the immune phenotype of Mtb-specific IFN-γ-producing CD4^+^ T cells in ATB and LTBI. We showed that compared to individuals with LTBI, PBMCs from ATB patients harbored significantly higher frequencies of Mtb-specific IFN-γ^+^CD4^+^ T cells expressing immune activation markers CD38 and HLA-DR and the intracellular proliferation marker Ki-67 (7). These markers accurately identified ATB patients and correlated with response to anti-TB treatment (7). Our studies showed that activated Mtb-specific IFN-γ^+^ producing CD4^+^ T cells can serve as an indirect gauge of Mtb antigen load within individuals. In this study, we extend the concept of antigen-specific T cell phenotypes as readouts of pathogen burden and investigate the expression of active caspase-3 in individuals with ATB and LTBI. Caspase-3, a member of the caspase family of cysteine proteases is expressed in CD4 effector T cells downstream of anti-CD3-mediated T cell receptor (TCR) activation (8) and has been shown to orchestrate apoptotic pathways during microbial infection following T cell activation and regulate T cell activation, cell cycle entry, proliferation, and differentiation (8–12). Since ATB patients have higher frequencies of activated Mtb-specific CD4^+^ T cells compared to LTBI, we hypothesized that ATB would also harbor higher frequencies of Mtb-specific CD4^+^ T cells expressing active caspase-3.

Using polychromatic flow cytometry, we evaluated the expression of active caspase-3 in Mtb-specific CD4^+^ T cells from ATB patients and individuals with LTBI. We found significantly higher frequencies of active caspase-3^+^IFN-γ^+^CD4^+^ T cells in ATB compared to LTBI. Further, caspase-3-expressing IFN-γ^+^CD4^+^ T cells were more activated compared to their caspase-3-negative counterparts and the frequencies of caspase-3^+^IFN-γ^+^CD4^+^ T cells decreased following successful anti-TB treatment, indicating that caspase-3 expression in Mtb-specific IFN-γ^+^CD4^+^ T cells is associated with mycobacterial burden.

## Materials and Methods

### Study Participants

This study was conducted according to the principles expressed in the Declaration of Helsinki. Ethical approval was obtained from the Emory University Institutional Review Board. All participants were provided written informed consent for the collection of samples and subsequent analyses. HIV-negative subjects between 23 and 83 years of age with LTBI (*n* = 23) or with pulmonary ATB disease (*n* = 22) were recruited in Atlanta, GA, USA. The 22 patients with confirmed pulmonary ATB were enrolled at Grady Memorial Hospital (Atlanta, GA, USA), prior to initiation of anti-TB treatment. Diagnosis of pulmonary ATB was based on the presence of clinical symptoms, sputum positivity by acid-fast bacilli (AFB) smear, positive amplified *Mycobacterium Tuberculosis* Direct assay, and positive culture (Table 1). All the ATB patients underwent the anti-TB treatment. However, only eight patients receiving the standard regimen of anti-TB treatment were followed longitudinally for 6 months. Anti-TB treatment was provided according to Centers for Disease Control (CDC) guidelines (13) for drug-susceptible TB and consisted of 2 months of isoniazid, rifampicin, pyrazinamide, and ethambutol, followed by 4 months of isoniazid and rifampicin. Resolution of TB was assessed by clinical, radiological, and microbiological criteria as described in Table 1. Healthy subjects from Atlanta, GA were identified as having LTBI by a positive ESAT6-CFP10-specific IFNγ-ELISPOT assay as described previously (14). These individuals were all HIV negative, non-smokers with no recent history of severe respiratory disease and had a normal chest X-ray. The presence of IFN-γ^+^CD4^+^ T cells in PBMCs from both ATB and LTBI groups was assessed by flow cytometry and intracellular cytokine staining (ICS) following stimulation with Mtb-CW antigens and ESAT6 and CFP10 peptide pools.

**Table 1:** Clinical characteristics of enrolled participants.

	Active tuberculosis	Latent *Mycobacterium tuberculosis* infection
Participants, *n*	22	23
Male, *n* (%)	20 (91%)	14 (61%)
Median age	53.5 (23–83) years	34.0 (22–61) years
Black race, *n* (%)	19 (86%)	8 (35%)
Culture proven pulmonary, *n* (%)	22 (100%)	Not done
ESAT6-CFP10 responders, *n* (%)	22 (100%)	23 (100%)
HIV seropositive, *n* (%)	0 (0%)	0 (0%)

### PBMC Isolation, Antigens, and Peptides for Cell Stimulations

Blood samples were collected from all subjects at baseline and longitudinal time points. PBMCs were isolated from blood as described previously (14) using cell preparation tubes (CPT, BD Biosciences) and cryopreserved in 90% fetal FBS (Hyclone, South Logan, UT) and 10% dimethyl sulfoxide (Sigma-Aldrich, St. Louis, MO, USA). PBMCs were stimulated with Mtb cell wall (CW) antigens (NIH-TBVRM contract, BEI) and ESAT6-CFP10 peptides pools, which were composed of 15-mers with 11 amino-acid overlap (Genemed Synthesis Inc., San Antonio, TX, USA).

### Flow Cytometry and Staining

For ICS, cryopreserved PBMCs were rested overnight at 37°C, 5% CO_2_ in RPMI-1640 medium (Lonza, Walkersville, MD, USA) containing 10% FBS, 2 mM glutamine, 100 IU/ml penicillin, and 100 µg/ml streptomycin. The viability of the lymphocytes was 75–95%. 1–2 × 10^6^ PBMCs were each stimulated with Mtb CW antigens (10 µg/ml; BEI Resources) and ESAT-6 and CFP-10 peptide pools (10 µg/ml) for 2 h followed by the addition of Brefeldin A (10 µg/ml) (BD Biosciences, San Diego, CA, USA) and further incubated for 16 h. PBMCs were stained for dead cells with the LIVE/DEAD Fixable Yellow Dead Cell Stain (Life Technologies, OR) at the beginning, and then surface-stained with appropriate antibodies: CD4 PerCp-Cy5.5 (clone L200), CD8 V500 (clone SK1), HLA-DR PE-Cy7 (clone L243), all from BD Biosciences, CD38 ECD (clone LS198.4.3) from Beckman-Coulter (Fullerton, CA, USA), CD45RA BV711 (clone HI100) from Biolegend (San Diego, CA, USA); permeabilized with Cytofix/Cytoperm Kit (BD Biosciences), stained intracellularly with appropriate antibodies: active caspase-3 FITC (clone C92-605), IFN-γ Alexa Fluor 700 (clone B27), IL-2-APC (clone MQ1-17H12), Ki-67 PE (clone B56), and CD3 APC-H7 (clone UCHT1), all from BD Biosciences, TNF-α BV650 (Clone MAb11, Biolegend); and fixed with 1% paraformaldehyde before acquisition on an LSR-II flow cytometry (BD Biosciences). Flow cytometry data were analyzed with FlowJo software (Tree Star Inc., San Carlos, CA, USA). Positive Mtb-specific CD4^+^ T cell responses were defined by a frequency of CD4^+^IFN-γ^+^ T cells of ≥0.05%. The minimum number of CD4^+^IFN-γ^+^ T cells used in this study to assess caspase-3 expression was 175 events.

### Statistical Analysis

Data were analyzed using Graphpad Prism 6.0b software. The Mann–Whitney *U* test was used to compare two groups. The means was used for descriptive statistics for each parameter. Differences between paired samples were analyzed using the Wilcoxon matched-paired rank test. A *P*-value of less than 0.05 was considered to be statistically significant.

## Results

### Expression of Caspase-3 in Mtb-Specific CD4^+^ T Cells in Individuals With ATB and LTBI

**Figure 1:**
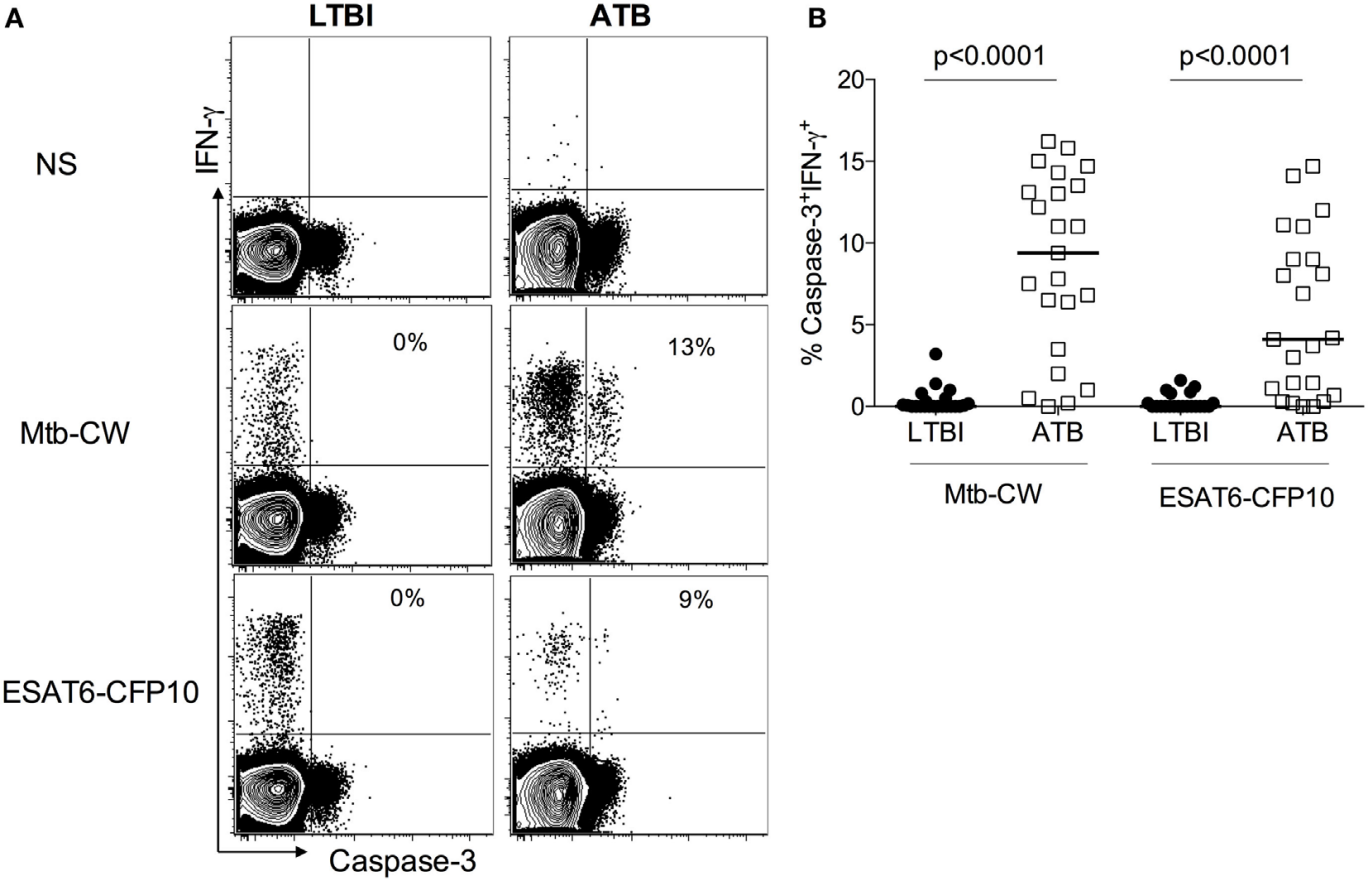
Caspase-3 expression on *Mycobacterium tuberculosis* (Mtb)-specific IFN-γ^+^CD4^+^ T cells differentiates between active tuberculosis (ATB) and latent Mtb infection (LTBI). Peripheral blood mononuclear cells from individuals with LTBI and ATB were stimulated with Mtb cell wall antigens and ESAT6-CFP10 peptide pools or non-stimulated (NS) **(A)** Representative flow plots for one active tuberculosis (ATB) and one LTBI individual and **(B)** cumulative data for ATB (*n* = 22) and LTBI (*n* = 23) groups. The frequencies of caspase-3^+^IFN-γ^+^ T cells were compared between LTBI and ATB groups. Mann–Whitney *U* test was used to compare the two groups. A *P*-value of less than 0.05 was considered to be statistically significant. Bars represent means.

Gating on live lymphocytes (Figure S1 in Supplementary Material), we evaluated the expression of active caspase-3 on IFN-γ^+^CD4^+^ T cells in 22 ATB patients and 23 healthy subjects with LTBI (Table 1), after stimulating PBMCs with Mtb-CW antigens and ESAT6-CFP10 peptides pools. A representative flow cytometry plot from one ATB patient and one LTBI subject (Figure 1A) and the summarized data (Figure 1B) show that ATB patients harbor significantly higher frequencies of Mtb-specific IFN-γ^+^CD4^+^ T cells expressing caspase-3 compared to LTBI (9.1 vs 0.1, *p* < 0.0001 with Mtb CW antigens; 4.1 vs 0.1, *p* < 0.0001 with ESAT6-CFP10 peptides pools). These differences were restricted to antigen-specific IFN-γ^+^CD4^+^ T cells as expression of caspase-3 in bulk, non-stimulated populations of CD4^+^ T cells was similar in both groups (Figure S2 in Supplementary Material).

### Caspase-3^+^IFN-γ^+^CD4^+^ T Cells in ATB Patients Display an Activated Effector Phenotype

**Figure 2:**
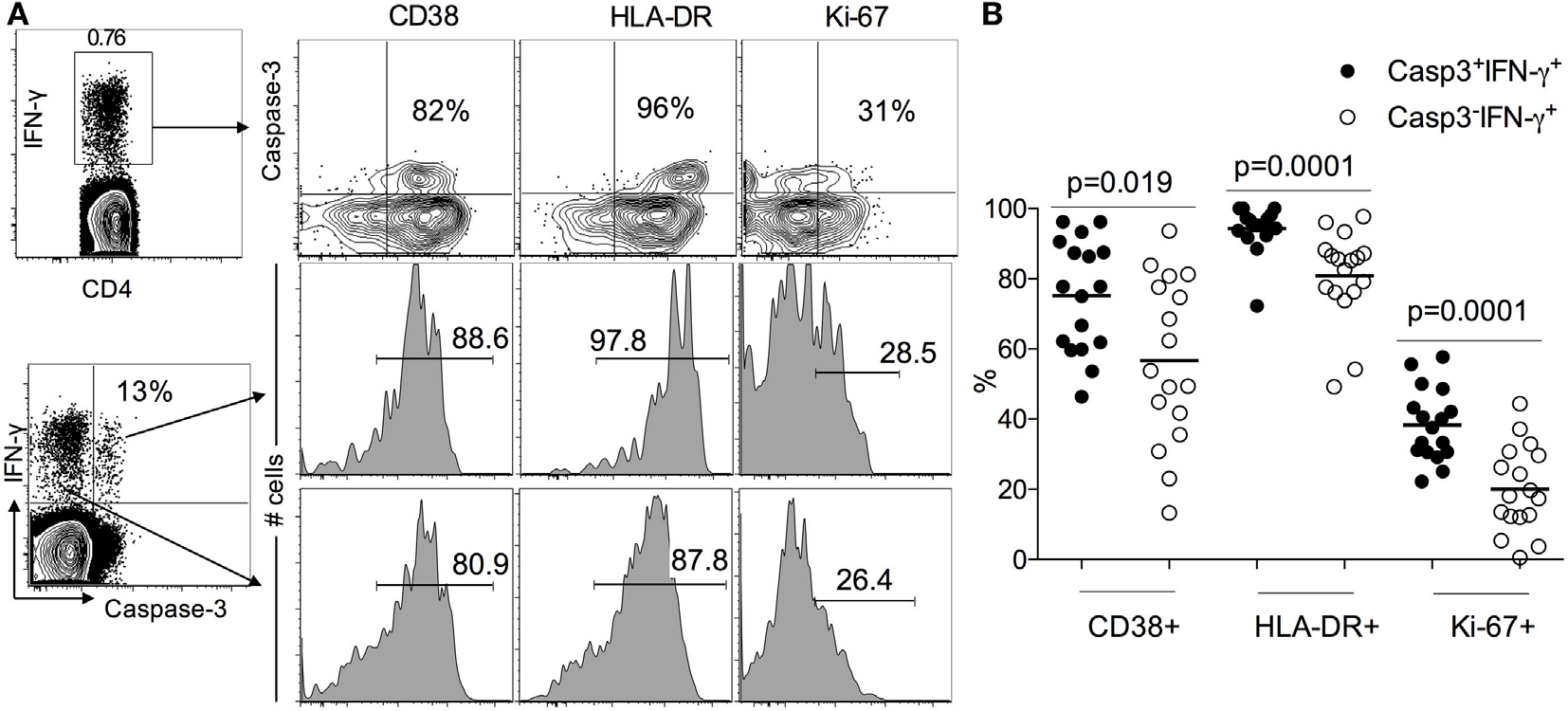
Caspase-3^+^IFN-γ^+^CD4^+^ T cells are more activated than caspase-3^−^IFN-γ^+^CD4^+^ T in ATB patients. **(A)** Representative flow plots for one patient and **(B)** cumulative data for 17 patients. The frequencies of immune activation markers CD38, HLA-DR, and the intracellular proliferative marker Ki-67 were compared between caspase-3^+^IFN-γ^+^ and caspase-3^−^ IFN-γ^+^CD4^+^ T cells. Mann–Whitney *U* test was used to compare the two groups. A *P*-value of less than 0.05 was considered to be statistically significant. Bars represent means.

**Figure 3:**
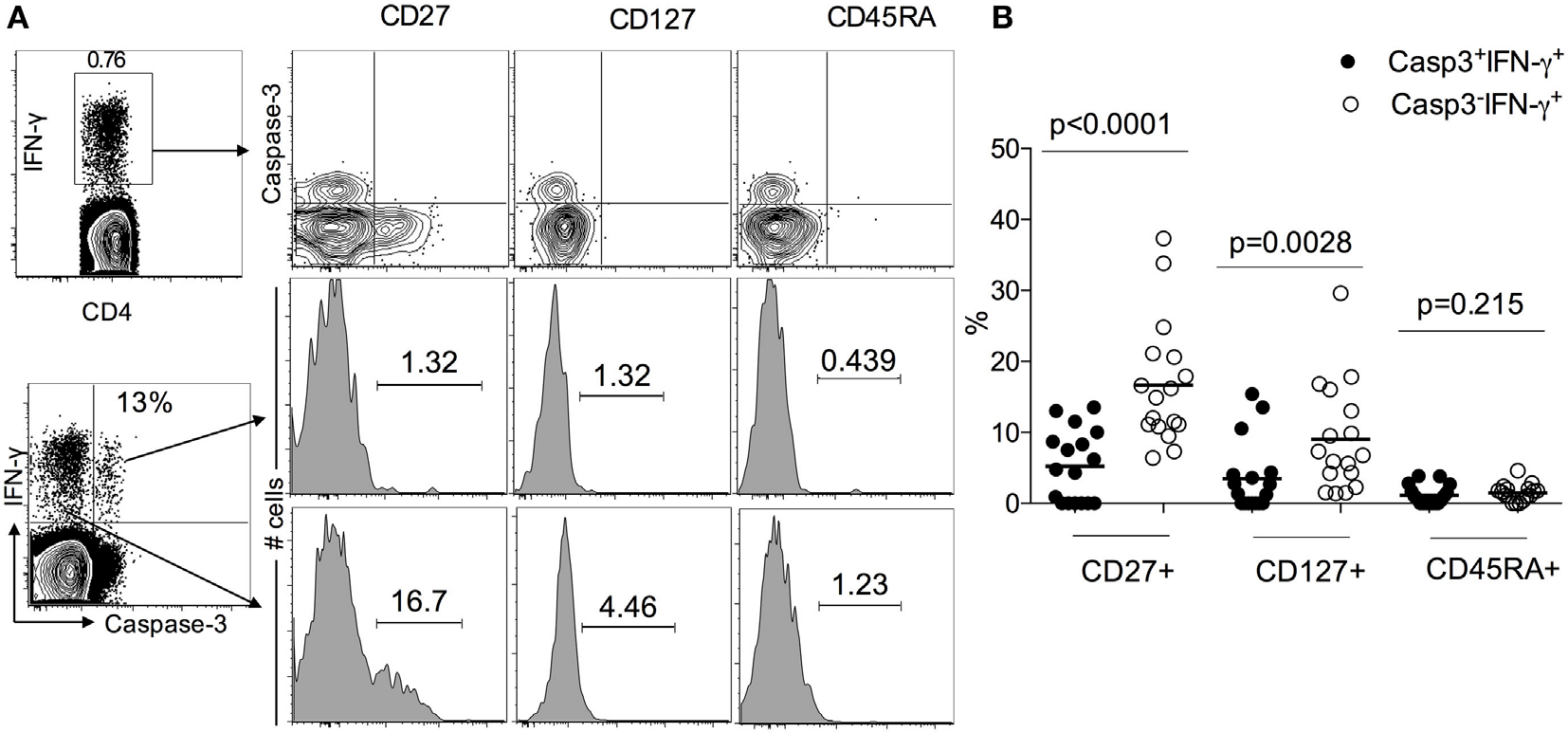
Caspase-3^+^IFN-γ^+^CD4^+^ T cells display more effector phenotype than caspase-3^−^ IFN-γ^+^CD4^+^ T in active tuberculosis patients. **(A)** Representative flow plots for one patient and **(B)** cumulative data for 17 patients. The frequencies of the memory differentiation markers CD27, CD127, and CD45RA were compared between caspase-3^+^IFN-γ^+^ and caspase-3^−^IFN-γ^+^CD4^+^ T cells. Mann–Whitney *U* test was used to compare the two groups. A *P*-value of less than 0.05 was considered to be statistically significant. Bars represent means.

Since LTBI individuals do not express caspase-3^+^IFN-γ^+^CD4^+^ T cells (Figure 1), to assess the activation state of caspase-3^+^IFN-γ^+^CD4^+^ T cells in ATB, we examined the expression of immune activation markers CD38, HLA-DR, and the intracellular proliferative marker Ki-67 in caspase-3^+^IFN-γ^+^ and caspase-3^−^IFN-γ^+^CD4^+^ T cells. We found that caspase-3^+^IFN-γ^+^CD4^+^ T cells express higher levels of CD38 (75 vs 55%, *p* = 0.019), HLA-DR (98 vs 80%), and Ki-67 (40 vs 20%) compared to caspase-3^−^IFN-γ^+^CD4^+^ T cells (Figure 2) upon stimulation with Mtb-CW antigens. These data show that the expression of caspase-3 in Mtb-specific CD4^+^ T cells is associated with an activated cycling state. To assess the differentiation state associated with caspase-3^+^IFN-γ^+^CD4^+^ T cells, we analyzed the expression of CD27, CD45RA, and CD127. Caspase-3^+^IFN-γ^+^CD4^+^ T cells were characterized by mostly CD27^−^CD45RA^−^CD127^−^ cells indicating an effector phenotype (Figure 3).

### Reduced IL-2 Production in Mtb-Specific Caspase-3^+^IFN-γ^+^ Compared to Caspase-3^−^IFN-γ^+^CD4^+^ T Cells in ATB Patients

**Figure 4:**
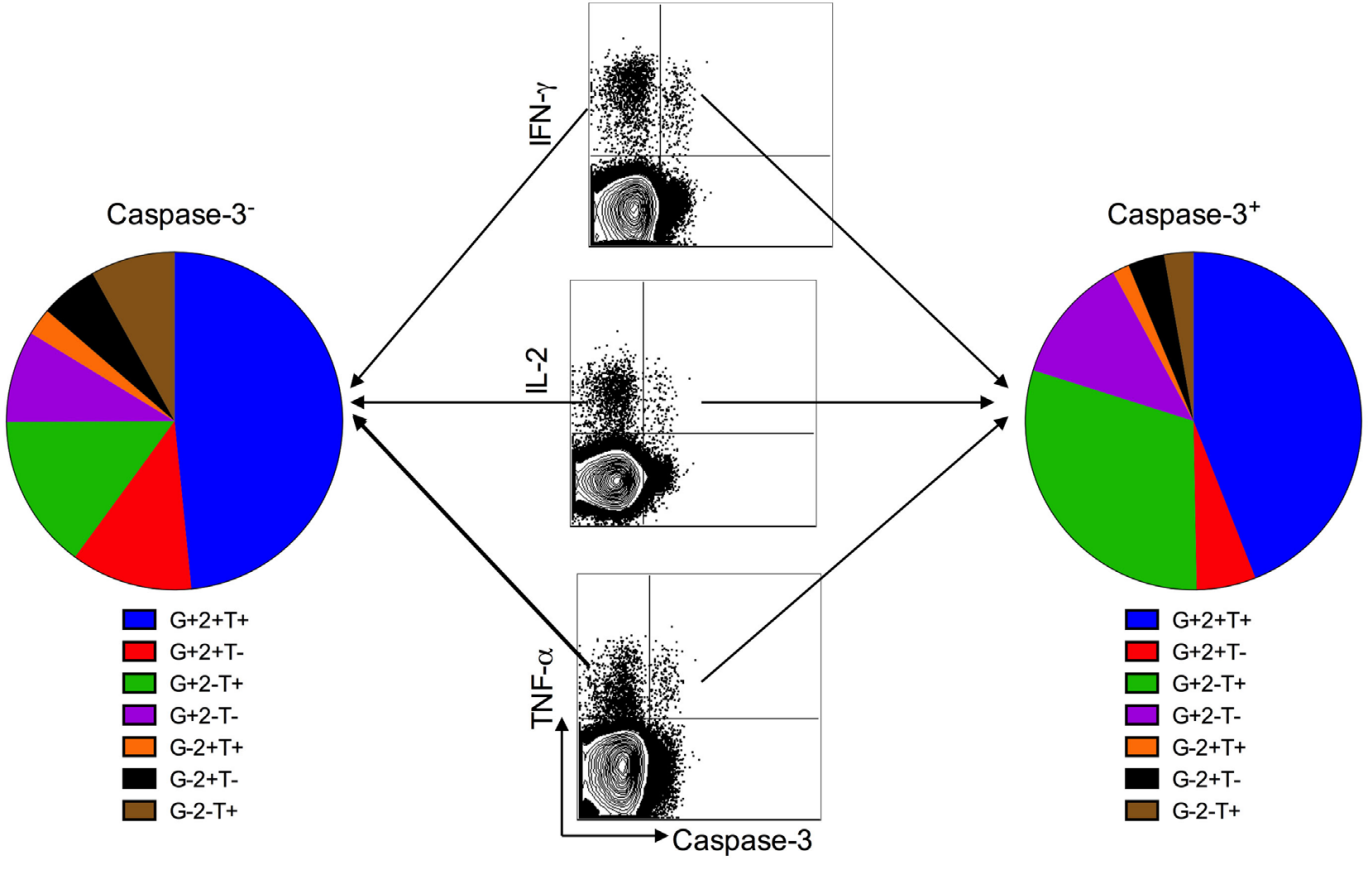
Caspase-3^+^ and caspase-3^−^ antigen-specific CD4^+^ T cell subsets are polyfunctional. Polyfunctional cytokine responses from caspase-3^+^ and caspase-3^−^ antigen-specific CD4^+^ T cell subsets in active tuberculosis patients. Data are represented as the percentage of responding CD4^+^ T cells that are triple producers, double producers, or single producers of IFN-γ (G), TNF-α (T), and IL-2 (2) and summarized by the pie charts. Each slice of the pie represents the fractions of the total response that consists of CD4^+^ T cells positive for a given function.

**Figure 5:**
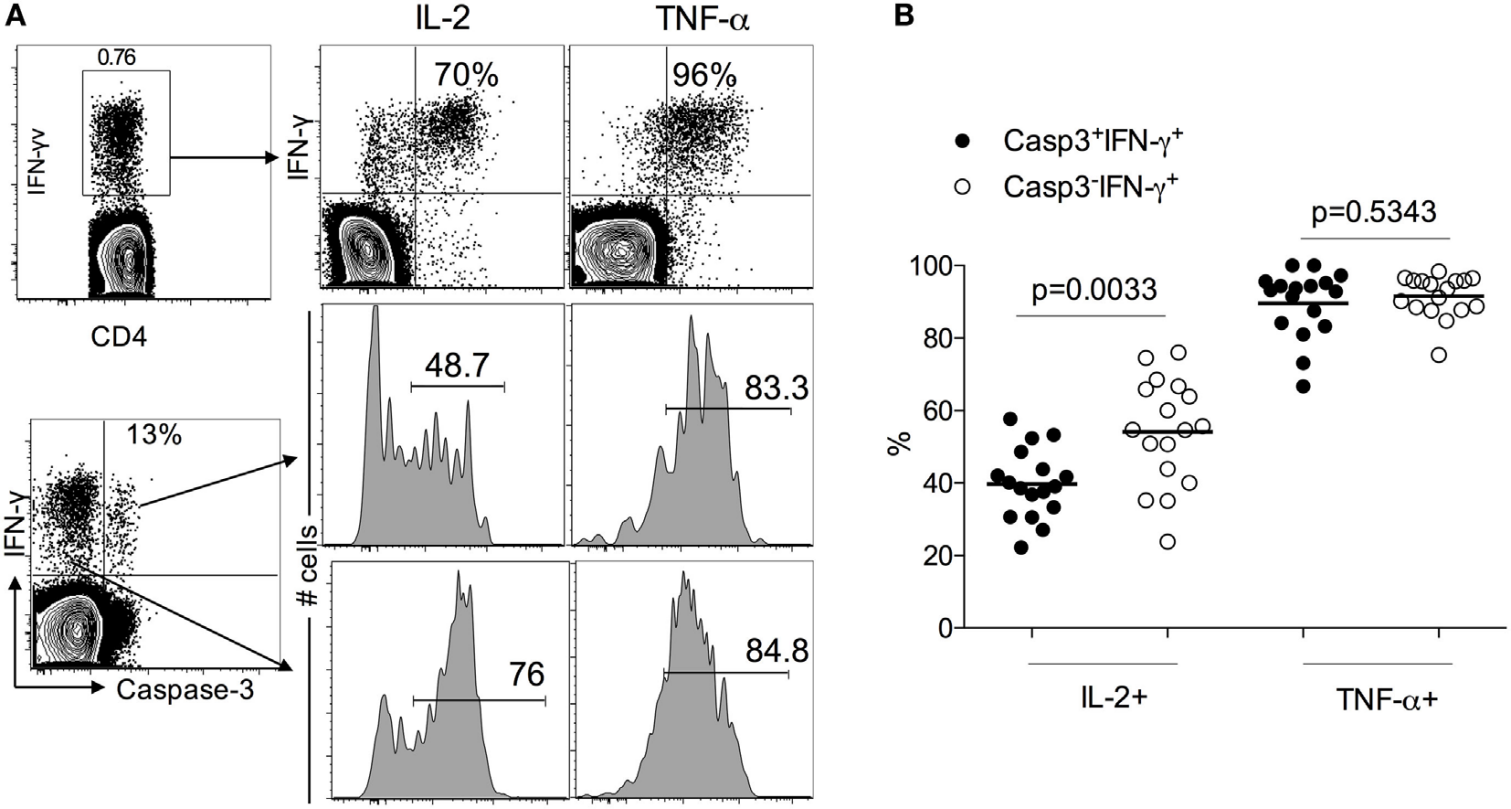
IL-2 and TNF-a expression caspase-3^+^ and caspase-3^−^ antigen-specific CD4^+^ T cell subsets in active tuberculosis patients. **(A)** Representative flow plots for one patient and **(B)** cumulative data for 17 patients.

Several studies have demonstrated the presence of functional Mtb-specific CD4^+^ T cell responses in treatment-naïve pulmonary TB patients by assessing the production of IL-2, TNF-α, and IFN-γ in PBMCs stimulated with Mtb antigens. To evaluate the functionality of caspase 3-expressing Mtb-specific CD4 T cells, we investigated the expression of IL-2 and TNF-α in caspase-3^+^IFN-γ^+^ and caspase-3^−^IFN-γ^+^ CD4 T cell subsets. Both caspase-3^+^ and caspase-3^−^ subsets exhibited polyfunctional responses, as seen by their capacity to produce IFN-γ, TNF-α, and IL-2 in response to Mtb antigen stimulation (Figure 4). However, caspase-3^+^IFN-γ^+^CD4^+^ T cells exhibited lower levels of IL-2 compared to caspase-3^−^IFN-γ^+^CD4^+^ T cells (40 vs 55%, *p* = 0.003; Figures 5A,B). Thus, caspase-3 expression in Mtb-specific CD4^+^ T cells in ATB is associated with reduced IL-2 levels.

### Frequencies of Mtb-Specific Caspase-3^+^IFN-γ^+^ CD4 T Cells Decrease Following Successful Anti-TB Treatment

**Figure 6:**
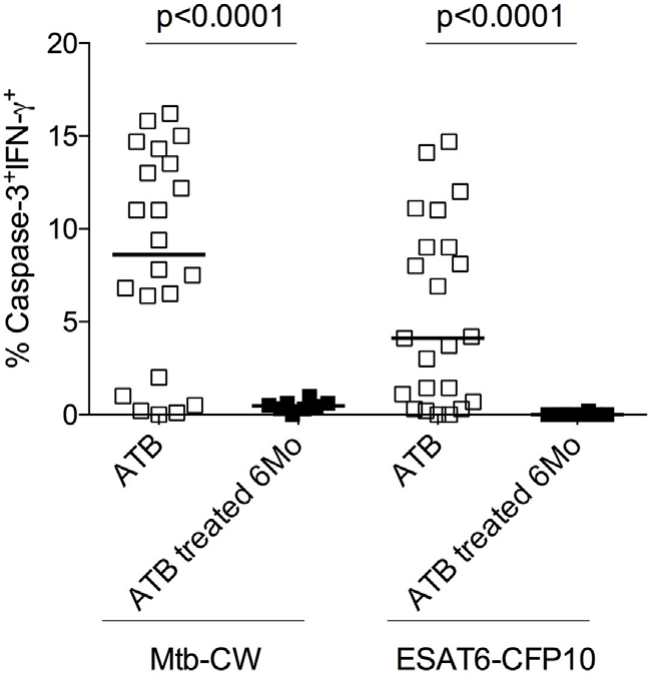
Frequencies of caspase-3^+^ *Mycobacterium tuberculosis* (Mtb)-specific CD4^+^ T cells at baseline and 6 months after anti-TB treatment. Analysis of the frequencies of caspase-3^+^IFN-γ^+^CD4^+^ T cells in individuals with treatment-naive active tuberculosis (ATB) (*n* = 23) as well as those who received 6 months of anti-TB treatment (ATB treated 6 months; *n* = 8). Mann–Whitney *U* test was used to compare the two groups. A *P*-value of less than 0.05 was considered to be statistically significant. Bars represent means.

**Figure 7:**
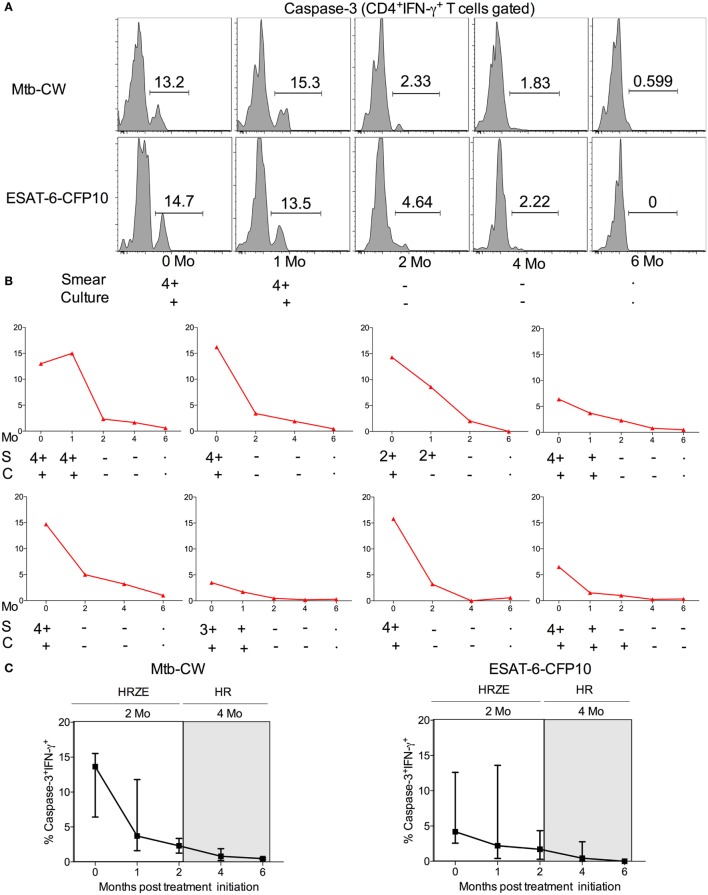
Comparison of the frequencies of caspase-3^+^IFN-γ^+^CD4^+^ T cells in 7 TB patients undergoing the 6-month regimen of anti-TB treatment. **(A)** Representative flow plots for one patient, **(B)** frequencies of caspase-3^+^IFN-γ^+^CD4^+^ T cells in eight different ATB patients over the course of anti-TB treatment after stimulation with Mtb-CW, and **(C)** cumulative data for eight patients are shown for the frequencies of caspase-3^+^IFN-γ^+^ T cells. The 2-month intensive phase (HRZE) and 4-month continuation phase (HR) are indicated. Abbreviations: S = sputum smear, C = culture.

The standard treatment regimen for TB consists of a 2-month intensive phase with isoniazid, rifampicin, pyrazinamide, and ethambutol (HRZE) followed by a month-continuation phase with isoniazid and rifampicin (HR) (15). To investigate whether frequencies of caspase-3^+^IFN-γ^+^CD4^+^ T cells in treatment-naive ATB patients are altered following anti-TB treatment, we compared treatment-naïve ATB patients with those who successfully completed 6 months of anti-TB treatment (*n* = 8) by assessing frequencies of caspase-3^+^IFN-γ^+^CD4^+^ T cells in PBMCs stimulated with Mtb-CW and ESAT6-CFP10. The frequencies of caspase-3^+^IFN-γ^+^CD4^+^ T cells were significantly lower in the treated ATB group (ATB treated-6 months) compared with untreated ATB group (Figure 6). We next assessed caspase-3^+^IFN-γ^+^ CD4^+^ T cell frequencies at baseline (time 0) and at multiple time points after treatment initiation for a subset of individuals for whom we had longitudinal samples (*n* = 8). Baseline diagnosis by sputum AFB smear and culture is indicated for each patient, and sputum was monitored for AFB smear and culture conversion during treatment (Figure 7). Conversion to a negative sputum culture at 2 months after the initiation is currently the most objective indicator of response to treatment (15). Caspase-3^+^IFN-γ^+^CD4^+^ T cells were relatively prevalent until 30–60 days after anti-TB treatment, after which their levels were greatly reduced (Figures 7A–C). Overall, these data show that, compared to treatment naive ATB patients, the frequencies of caspase-3^+^IFN-γ^+^CD4^+^ T cells were significantly reduced after successful completion of the 6-month standard regimen of anti-TB treatment (Figures 6 and 7). These data suggest that reduction in mycobacterial burdens during successful anti-TB treatment correlates with reduced frequencies of caspase-3 in Mtb-specific IFN-γ^+^CD4^+^ T cells.

## Discussion

Several studies have demonstrated that the immune phenotypes of antigen-specific T cells, including the expression of activation markers, their maturation, and differentiation states and cytokine profiles, generally reflects the antigen burden within individuals infected with viral and bacterial pathogens (7, 16–18). Distinct T cell phenotypes have been associated with active pulmonary TB, where antigen burdens are typically high, compared to persistent or chronic infections (LTBI) with low antigenic burdens or after treatment-induced clearance of infection (7, 14, 19–28). We previously showed that frequencies of Mtb-specific IFN-γ^+^CD4^+^ T cells expressing each of the immune activation markers CD38 and HLA-DR and the intracellular proliferation marker Ki-67, accurately classified ATB and LTBI status and correlated with decreasing mycobacterial loads during treatment (7). In this study, we extended the concept of antigen-specific T cell phenotypes as indirect readouts of pathogen burden and investigated the expression of active caspase-3 in activated Mtb-specific CD4^+^ T cells. Caspase-3 is known to be a major executor of apoptosis in antigen-stimulated T cells (29). However, several reports have suggested that caspase-3 might have an additional role in the immune system by promoting lymphocyte activation and proliferation (8–11, 30). For example, in studies on acute LCMV infection in mice, caspase-3 mRNA levels were shown to be selectively increased in peripheral T cells after antigen-specific stimulation (9) or following TCR stimulation (31). It has also been shown that caspase-3 is expressed in non-apoptotic T lymphocytes (32–34). In this study and gating on live lymphocytes, we showed higher frequencies of caspase-3-expressing Mtb-specific IFN-γ^+^CD4^+^ T cells in individuals with ATB compared to LTBI subjects, suggesting that the caspase-3 pathway is operant during active TB phase but not during the latent phase. We also showed that the caspase-3-expressing Mtb-specific IFN-γ^+^CD4^+^ T cells express high levels of T cell activation markers CD38 and HLA-DR and the proliferation marker Ki-67 (Figure 2), suggesting that caspase-3 expression is associated with activation and proliferation of CD4^+^ T cells during active TB disease. Indeed, caspase-3 has been shown to be involved in regulating early steps of lymphocyte activation, cell cycle entry, and proliferation (8, 35). The importance of caspases in T cell activation is also highlighted by defective T cell activation observed in humans lacking functional caspase-8, and caspase blockers have been shown to inhibit human T cells proliferation in response to various antigen stimulations (36, 37). It has recently been shown that the activation profile of Mtb-specific CD4^+^ T cells as assessed by the expression of CD38, HLA-DR, and Ki-67 reflects TB disease in both HIV infected and uninfected individuals (24). It is interesting to speculate that expression of caspase-3 in Mtb-specific CD4^+^ T cells from HIV-infected ATB patients will be similarly higher compared to HIV infected individuals with LTBI despite the reduction of peripheral CD4^+^ T cells during HIV infection (25, 38, 39).

Our observation of reduced IL-2 production in Mtb-specific caspase-3^+^ CD4^+^ T cells compared to caspase-3^−^ CD4^+^ T cells (Figure 5) suggests that caspase-3 expression may also be associated with reduced proliferative capacity in TB (19). Further, the functional differences between caspase-3^+^ and caspase-3^−^ CD4^+^ T cells suggested by this study may be explained by the maturation phenotype of the cells. Caspase-3-expressing Mb-specific CD4^+^ T cells exhibited a more differentiated effector T cell phenotype (CD27^−^CD45RA^−^CD127^−^) (Figure 3) in ATB patients (40–43). Expression of the costimulatory molecule CD27 on Mtb-specific T cells has been shown to identify ATB status in both adults (21, 26) and children (44). Whether caspase-3 expression in Mtb-specific T cells, along with CD38, HLA-DR, and Ki-67, will be useful as biomarkers for diagnosing TB in children needs further investigation.

A number of immunological markers measured in the blood before and after anti-TB treatment, have shown promising results as prognostic markers of clinical severity and/or predictors of microbiological outcome in TB patients (45–47). However, the majority of these studies have been focused on the plasma. Further, Berry et al. identified an IFN-inducible neutrophil-driven transcriptional signature that was associated with ATB disease and correlated with treatment response (48). We examined the effect of anti-TB treatment on the frequencies of caspase-3 expressing Mtb-specific IFN-γ^+^CD4^+^ T cells in active TB patients and found that treatment-mediated resolution was associated with the decline of antigen-specific caspase-3^+^IFN-γ^+^CD4^+^ T cells which correlate with Mtb burden *in vivo* using sputum smear and culture measurement (Figures 6 and 7). This is similar to what was reported in HIV-infected individuals where anti-retroviral therapy led to reduced frequencies of HIV-1-specific caspase-3 expressing CD4^+^ T cells (49). Overall, our studies show that frequencies of caspase-3 expressing antigen-specific CD4^+^ T cells may reflect mycobacterial burden *in vivo* and could, therefore, be useful for assessing anti-TB treatment response along with other host biomarkers (50–53).

## Ethics Statement

This study was conducted according to the principles expressed in the Declaration of Helsinki. Ethical approval was obtained from the Emory University Institutional Review Board. All participants were provided written informed consent for the collection of samples and subsequent analyses.

## Author Contributions

TA and JR conceived the study design. TA, SC, and SR recruited cohorts that were used in this study. TA, CI, SR, and JR acquired and/or analyzed and interpreted the data. TA and JR wrote the manuscript with assistance from CI. All authors reviewed, revised, and approved the manuscript for submission.

## Conflict of Interest Statement

The authors declare that the research was conducted in the absence of any commercial or financial relationships that could be construed as a potential conflict of interest.

## Supplementary Material

Figure S1 Representative gating strategy derived from total peripheral blood mononuclear cells (PBMCs): Cell doublets were excluded from the total population of PBMCs using forward scatter area (FSC-A) and height (FSC-H); the lymphocyte population was gated according to FSC-A and size scatter area, the dead lymphocytes were excluded using live-dead staining; CD3^+^ T cells were gated, followed by CD4^+^ T cells gating to assess the IFN-γ^+^ T cells.

Figure S2 Caspase-3 expression on bulk CD4^+^ T cells from latent Mtb infection (LTBI) and treatment-naïve active tuberculosis (ATB). **(A)** Representative flow plots for one ATB and one LTBI individual and **(B)** cumulative data for ATB (*n* = 22) and LTBI (*n* = 23) groups. Data were derived from non-stimulated peripheral blood mononuclear cells. Mann–Whitney *U* test was used to compare the two groups. A *P*-value of less than 0.05 was considered to be statistically significant.
