# Supplementary figures and images for: High Frequencies of Caspase-3 Expressing Mycobacterium tuberculosis-Specific CD4+ T Cells Are Associated With Active Tuberculosis
Source: Front Immunol. 2018 Jun 25;9:1481. doi: 10.3389/fimmu.2018.01481 (PMC6026800; doi:10.3389/fimmu.2018.01481)

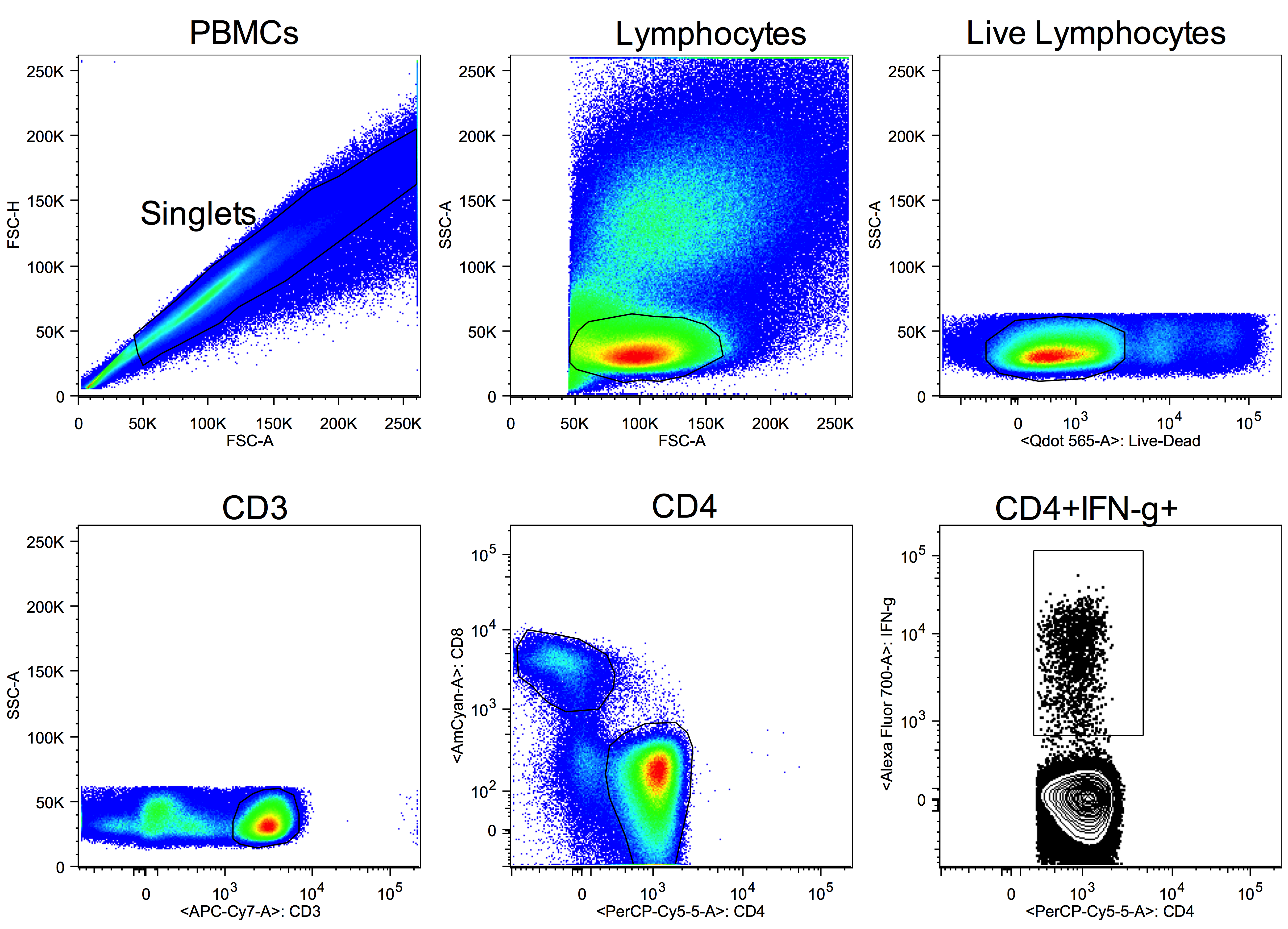

Supplement: Figure S1 — Representative gating strategy derived from total peripheral blood mononuclear cells (PBMCs): Cell doublets were excluded from the total population of PBMCs using forward scatter area (FSC-A) and height (FSC-H); the lymphocyte population was gated according to FSC-A and size scatter area, the dead lymphocytes were excluded using live-dead staining; CD3+ T cells were gated, followed by CD4+ T cells gating to assess the IFN-γ+ T cells. [file image_1.tiff]

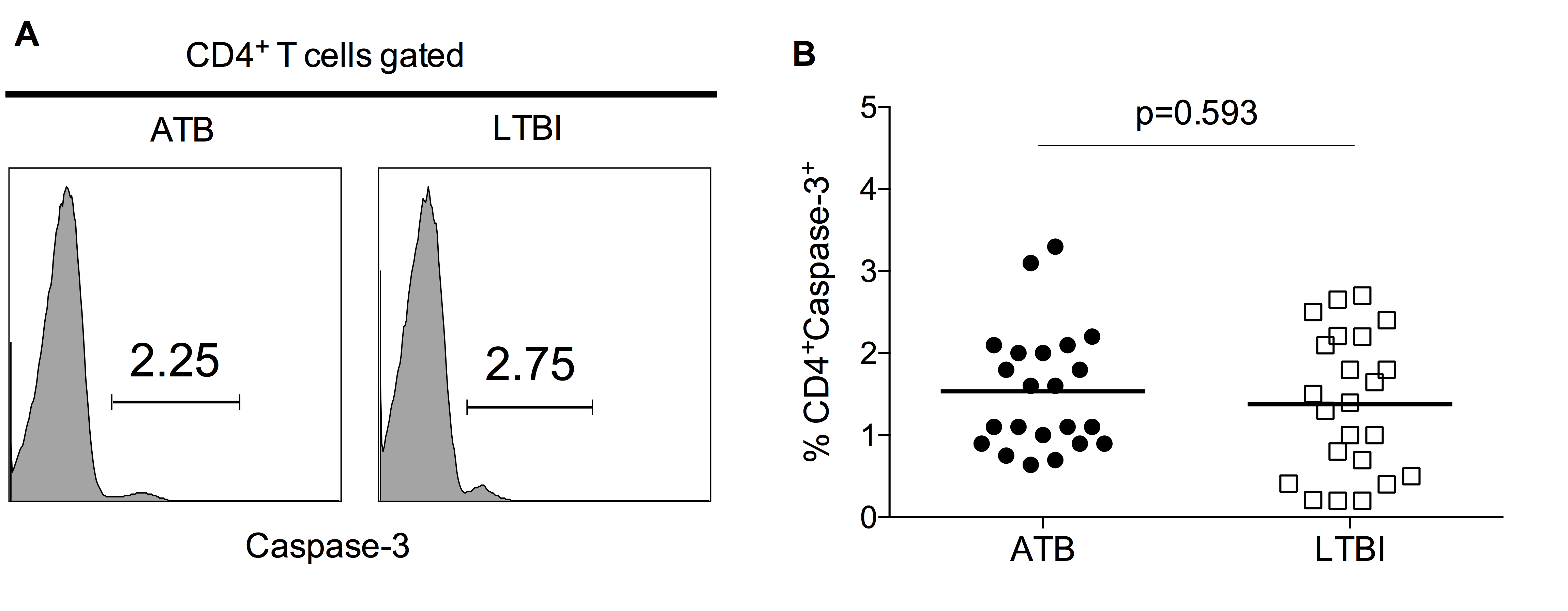

Supplement: Figure S2 — Caspase-3 expression on bulk CD4+ T cells from latent Mtb infection (LTBI) and treatment-naïve active tuberculosis (ATB). (A) Representative flow plots for one ATB and one LTBI individual and (B) cumulative data for ATB (n = 22) and LTBI (n = 23) groups. Data were derived from non-stimulated peripheral blood mononuclear cells. Mann–Whitney U test was used to compare the two groups. A P-value of less than 0.05 was considered to be statistically significant. [file image_2.tiff]
